# Syngeneic hematopoietic stem cell transplantation for acute myeloid leukemia: a propensity score-matched analysis

**DOI:** 10.1038/s41408-021-00553-w

**Published:** 2021-09-24

**Authors:** Shuhei Kurosawa, Shohei Mizuno, Yasuyuki Arai, Masayoshi Masuko, Junya Kanda, Kentaro Kohno, Daishi Onai, Takahiro Fukuda, Yukiyasu Ozawa, Yuta Katayama, Masatsugu Tanaka, Kazuhiro Ikegame, Naoyuki Uchida, Tetsuya Eto, Shuichi Ota, Junji Tanaka, Tatsuo Ichinohe, Yoshiko Atsuta, Masamitsu Yanada

**Affiliations:** 1 Division of Stem Cell and Molecular Medicine, The Institute of Medical Science, The University of Tokyo, Tokyo, Japan; 2 Division of Hematology, Department of Internal Medicine, Aichi Medical University, Nagakute, Japan; 3 Department of Hematology, Kyoto University Hospital, Kyoto, Japan; 4 Division of Stem Cell Transplantation, Niigata University Medical and Dental Hospital, Niigata, Japan; 5 Department of Hematology and Oncology, Graduate School of Medicine, Kyoto University, Kyoto, Japan; 6 Department of Hematology and Oncology, Japan Community Health Care Organization (JCHO) Kyushu Hospital, Kitakyushu, Japan; 7 Hematology Division, Tokyo Metropolitan Cancer and Infectious Diseases Center, Komagome Hospital, Tokyo, Japan; 8 Department of Hematopoietic Stem Cell Transplantation, National Cancer Center Hospital, Tokyo, Japan; 9 Department of Hematology, Japanese Red Cross Nagoya First Hospital, Nagoya, Japan; 10 Department of Hematology, Hiroshima Red Cross Hospital & Atomic-bomb Survivors Hospital, Hiroshima, Japan; 11 Department of Hematology, Kanagawa Cancer Center, Yokohama, Japan; 12 Department of Hematology, Hyogo College of Medicine Hospital, Nishinomiya, Japan; 13 Department of Hematology, Federation of National Public Service Personnel Mutual Aid Associations Toranomon Hospital, Tokyo, Japan; 14 Department of Hematology, Hamanomachi Hospital, Fukuoka, Japan; 15 Department of Hematology, Sapporo Hokuyu Hospital, Sapporo, Japan; 16 Department of Hematology, Tokyo Women’s Medical University, Tokyo, Japan; 17 Department of Hematology and Oncology, Research Institute for Radiation Biology and Medicine, Hiroshima University, Higashi-Hiroshima, Japan; 18 Japanese Data Center for Hematopoietic Cell Transplantation, Nagoya, Japan; 19 Department of Healthcare Administration, Nagoya University Graduate School of Medicine, Nagoya, Japan; 20 Department of Hematology and Cell Therapy, Aichi Cancer Center, Nagoya, Japan

**Keywords:** Acute myeloid leukaemia, Cancer immunotherapy

## Abstract

The present study evaluated outcomes and prognostic factors in adult patients with acute myeloid leukemia (AML) after syngeneic hematopoietic stem cell transplantation (HSCT). Among patients in first complete remission (CR1), outcomes of syngeneic HSCT (Syn) were compared with those of autologous HSCT (Auto), allogeneic HSCT from human leukocyte antigen (HLA)-matched sibling donor (MSD), or allogeneic HSCT from HLA-matched unrelated donor (MUD). Among 11,866 patients receiving first HSCT, 26 in the Syn group were analyzed. The 5-year overall survival (OS) rate, the cumulative incidence of relapse, and the cumulative incidence of non-relapse mortality (NRM) were 47.8%, 59.6%, and 4.6%, respectively. The OS was significantly better in patients in CR1 (*n* = 13) than in patients in non-CR1 (*P* = 0.012). Furthermore, 39 patients in CR1 each were assigned to the Auto, MSD, and MUD groups using propensity score matching. The 5-year OS in the Syn (68.4%) was not significantly different from those in the Auto (55.9%, *P* = 0.265), MSD (62.4%, *P* = 0.419), or MUD (63.7%, *P* = 0.409) groups. A higher relapse in the Syn than in the MSD and MUD groups was offset by lower NRM. In summary, syngeneic HSCT might be an alternative option for AML patients in CR1.

## Introduction

Allogeneic hematopoietic stem cell transplantation (HSCT) is a potentially curative therapeutic option for acute myeloid leukemia (AML) [1–7]. Although a human leukocyte antigen (HLA)-matched sibling donor (MSD) remains the best option, research to determine the next-best alternative is ongoing worldwide owing to the increasing number of available unrelated donors, cord blood units, and recent widespread use of haploidentical donors [1–4]. However, in the absence of an MSD, there is an increased risk of non-relapse morbidity and mortality owing to graft-versus-host disease (GVHD), severe infections, and regimen-related toxicities, which are the major obstacles to allogeneic HSCT. Autologous HSCT is an alternative post-remission treatment, which has been shown to reduce the risk of transplant-related morbidity and mortality [8–13]. However, a relatively high risk of relapse is a major problem owing to the lack of a graft-versus-leukemia effect by allogeneic cells and the potential contamination of the graft with leukemic cells.

Syngeneic HSCT is rarely performed in patients who have an identical twin [14–16]. Its effectiveness has not been fully understood in patients with AML. Previous studies to date have analyzed the outcomes of syngeneic HSCT for AML together with those for other hematological malignancies and non-hematological diseases, although they have different clinical characteristics [17–20]. In addition, limited data are available comparing the outcomes of syngeneic HSCT with those of autologous or allogeneic HSCT [17].

To clarify these issues, in the present study, we aimed to evaluate outcomes and prognostic factors in patients with AML after syngeneic HSCT and to compare these outcomes with those of autologous or allogeneic HSCT for patients in first complete remission (CR1) using the national registry data of the Transplant Registry Unified Management Program (TRUMP) in Japan.

## Materials and methods

### Data collection and study population

Clinical data were provided by the second-generation TRUMP of the Japanese Data Center for Hematopoietic Cell Transplantation [21, 22]. The TRUMP currently covers nearly all of the >300 transplantation centers nationwide in Japan, with a registration rate of >99%. Each participating institution is required to consecutively register anonymous information on all patients undergoing HSCT at their institution and to report follow-up information annually. The second-generation TRUMP, which was released in 2015, is a web-based HSCT registry database and can also be used offline for transplant centers not capable of reporting online. The study protocol complied with the principles of the Declaration of Helsinki. Approval for this retrospective study was obtained from the ethics committee of the University of Tokyo. Informed consent was obtained from each patient.

**Fig. 1:**
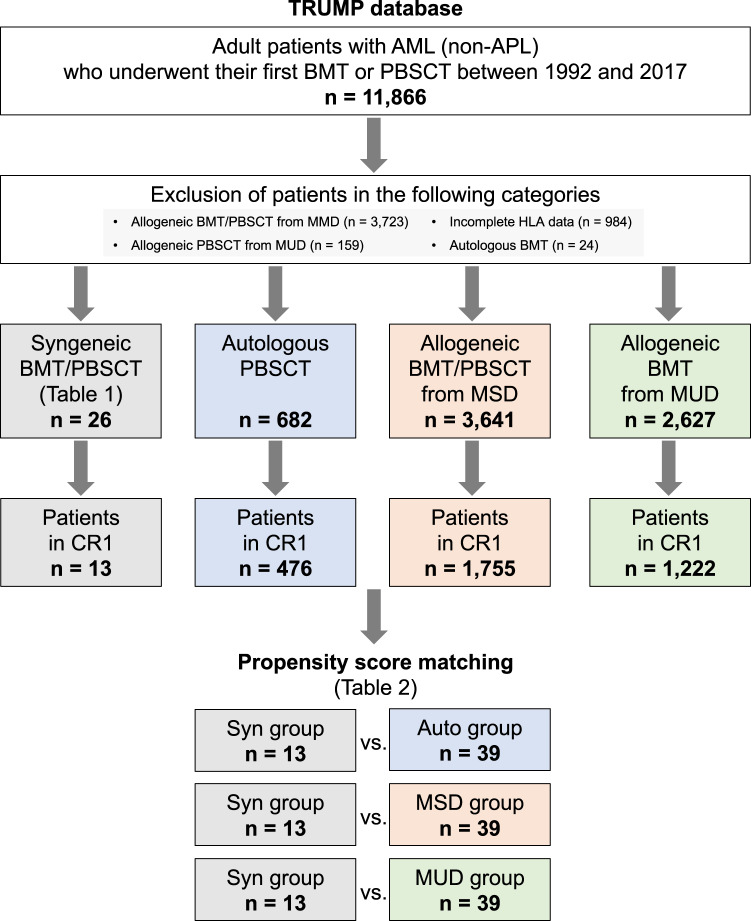
Patient selection flowchart and study design. *AML* acute myeloid leukemia, *APL* acute promyelocytic leukemia, *Auto* autologous, *BMT* bone marrow transplantation, *CR1* first complete remission, *HLA* human leukocyte antigen, *MMD* mismatched donor, *MSD* matched sibling donor, *MUD* matched unrelated donor, *PBSCT* peripheral blood stem cell transplantation, *Syn* syngeneic, *TRUMP* Transplant Registry Unified Management Program.

A flowchart of patient selection is shown in Fig. 1. Patients eligible for enrollment met the following criteria: (1) age ≥16 years; (2) diagnosis of AML (except for acute promyelocytic leukemia); and (3) receiving their first bone marrow transplantation (BMT) or peripheral stem cell blood transplantation (PBSCT) between 1992 and 2017. Among them, the following four groups were selected: a “Syn” group included those who underwent syngeneic BMT or PBSCT; “Auto” group, autologous PBSCT; “MSD” group, allogeneic BMT or PBSCT from an MSD; and “MUD” group, allogeneic BMT from an HLA-matched unrelated donor (MUD). Syngeneic HSCT was defined as a HSCT from a syngeneic identical twin [17–20]. We confirmed donor monozygosity based on the gender match, HLA, and ABO blood types registered in our database. HLA disparity was defined as a mismatch of at least one serologic level in related HSCT or allele level in unrelated HSCT detected between the recipient and donor. Patients receiving either allogeneic PBSCT from an unrelated donor (*n* = 159) or autologous BMT (*n* = 24) were not included because these procedures were rarely performed in Japan during the study period [6, 13]. Patients receiving either allogeneic BMT or PBSCT from an HLA-mismatched donor (*n* = 3723) or with incomplete HLA data (*n* = 984) were excluded from the present study.

### Study endpoints and definitions

The primary endpoint was the 5-year overall survival (OS) rate after HSCT. The secondary endpoints were the 5-year leukemia-free survival (LFS), 5-year cumulative incidence of relapse, 5-year cumulative incidence of non-relapse mortality (NRM), days from HSCT to neutrophil and platelet engraftment, 100-day cumulative incidence of acute GVHD, and 1-year cumulative incidence of chronic GVHD after HSCT.

OS was defined as the time from transplantation to death from any cause or last visit. LFS was defined as the time from transplantation to death, relapse, or last visit. Relapse was defined as the loss of CR in patients who at one time had achieved CR; meanwhile, patients who had never achieved CR after transplantation were categorized as relapse cases at time zero. CR was defined as the presence of <5% of blasts in the bone marrow (BM), absence of leukemic blasts in the peripheral blood or extramedullary sites, and recovery of blood counts. NRM was defined as death without relapse. Engraftment after HSCT was evaluated according to a conventional definition, as previously reported [23]. Acute and chronic GVHD were diagnosed and graded according to previously established criteria [24, 25]. The hematopoietic cell transplantation-specific comorbidity index was calculated, as described previously [26]. The cytogenetic risk was classified based on the published criteria [27].

### Statistical analysis

OS and LFS rates were estimated using the Kaplan–Meier method, and the log-rank test was used to assess significant differences. Cumulative incidence rates of relapse, NRM, acute GVHD, and chronic GVHD were evaluated using Gray’s method, considering NRM as a competing risk factor for relapse, relapse as a competing risk factor for NRM, and death or relapse as a competing risk factor for acute and chronic GVHD.

To evaluate factors influencing transplant outcomes in the Syn group, the following variables were evaluated in univariate analyses: age at HSCT (<40 years vs. ≥40 years), sex (female vs. male), cytogenetic risk (favorable vs. intermediate vs. poor vs. unevaluable), disease status at HSCT (CR1 vs. non-CR1), graft source (BM vs. peripheral blood stem cell [PBSC]), GVHD prophylaxis (administration of cyclosporin, tacrolimus, or methotrexate vs. no prophylaxis), and the year of HSCT (1992–2003 vs. 2004–2017).

To minimize selection bias and confounding factors, we performed propensity score (PS) matching analysis [28]. Among patients in CR1, PSs between the Syn and Auto, Syn and MSD, and Syn and MUD groups were calculated using logistic regression with the following factors: age at HSCT (<40 years vs. ≥40 years), sex (female vs. male), cytogenetic risk (favorable vs. intermediate vs. poor vs. unevaluable), and the year of HSCT (1992–2003 vs. 2004–2017). Graft source (BM vs. PBSC) was also used to calculate the PS between the Syn and MSD groups. Matching was performed using the nearest-neighbor matching method, with the caliper width fixed at 0.2. The ratio of the Syn group to the Auto, MSD, and MUD group was 1:3. The C-statistic was calculated to evaluate the discrimination of the PS. To compare baseline characteristics between the Syn and Auto, MSD, and MUD groups, categorical variables were compared using Fisher’s exact test. The balance of covariates after PS matching was assessed using *P* values and standardized mean differences.

All tests were two-sided, and *P* values <0.05 were considered statistically significant. A standardized mean difference <0.10 was considered to indicate a negligible difference between the PS-matched groups [29]. All statistical analyses were performed using EZR, a graphical user interface for R software (The R Foundation for Statistical Computing, version 4.0.2, Vienna, Austria) [30].

## Results

### Patient characteristics and transplant outcomes in the Syn group

Patient characteristics of the Syn group are summarized in Table 1. Overall, 26 patients were included in the Syn group. Among them, 13 (50.0%) patients were in CR1. Graft source was BM for 11 (42.3%) patients and PBSC for 15 (57.7%). Eight (30.8%) patients received GVHD prophylaxis, whereas 17 (65.4%) did not receive any GVHD prophylaxis with available data (*n* = 25 of 26, 96.2%). The median follow-up period for survivors was 3644 days (range, 315–9335 days).

**Table 1:** Characteristics of patients in the syngenic group.

	All patients		Patients in CR1	
Total number	26		13	
Age at HSCT				
Median	40	(18–55)	40	(18–55)
<40 years	11	(42.3%)	6	(46.2%)
≥40 years	15	(57.7%)	7	(53.8%)
Sex				
Female	8	(30.8%)	4	(30.8%)
Male	18	(69.2%)	9	(69.2%)
Performance status				
0–1	17	(65.4%)	11	(84.6%)
≥2	2	(7.7%)	0	(0.0%)
NA	7	(26.9%)	2	(15.4%)
HCT-CI				
0–1	11	(42.3%)	9	(69.2%)
≥2	1	(3.8%)	0	(0.0%)
NA	14	(56.0%)	4	(30.8%)
FAB subtypes				
M1	3	(11.5%)	2	(15.4%)
M2	14	(53.8%)	5	(38.5%)
M4	4	(15.4%)	3	(23.1%)
M5	2	(7.7%)	2	(15.4%)
M6	2	(7.7%)	1	(7.7%)
Others	1	(3.8%)	0	(0.0%)
Cytogenetic risk at diagnosis				
Favorable	5	(19.2%)	2	(15.4%)
Intermediate	15	(57.7%)	10	(76.9%)
Poor	3	(11.5%)	0	(0.0%)
Unevaluable	3	(11.5%)	1	(7.7%)
Disease status at HSCT				
CR1	13	(50.0%)	13	(100.0%)
CR2	4	(15.4%)		
CR3 or later	1	(3.8%)		
Non-CR	8	(30.8%)		
Time from diagnosis to HSCT				
Median	218	(27–2,885)	203	(128–429)
<240 days	14	(53.8%)	9	(69.2%)
≥240 days	11	(42.3%)	4	(30.8%)
NA	1	(3.8%)	0	(0.0%)
Graft source				
BM	11	(42.3%)	5	(38.5%)
PBSC	15	(57.7%)	8	(61.5%)
Conditioning				
BuCy	8	(30.8%)	5	(38.5%)
CyTBI	7	(26.9%)	4	(30.8%)
Other MAC	3	(11.5%)	0	(0.0%)
RIC	2	(7.7%)	1	(7.7%)
Others	6	(23.1%)	3	(23.1%)
GVHD prophylaxis				
Cyclosporine-based	6	(23.1%)	3	(23.1%)
Tacrolimus based	1	(3.8%)	0	(0.0%)
Others	1	(3.8%)	1	(7.7%)
No prophylaxis	17	(65.4%)	9	(69.2%)
NA	1	(3.8%)	0	(0.0%)
Year of HSCT				
1992–2003	13	(50.0%)	6	(46.2%)
2004–2017	13	(50.0%)	7	(53.8%)

*BM* bone marrow, *BuCy* busulfan and cyclophosphamide, *CR* complete remission, *CyTBI* cyclophosphamide and total body irradiation, *FAB* French American British, *GVHD* graft-versus-host disease, *HCT-CI* hematopoietic cell transplantation-specific comorbidity index, *HSCT* hematopoietic stem cell transplantation, *MAC* myeloablative conditioning, *NA* not available, *PBSC* peripheral stem cell blood, *RIC* reduced-intensity conditioning.

**Fig. 2:**
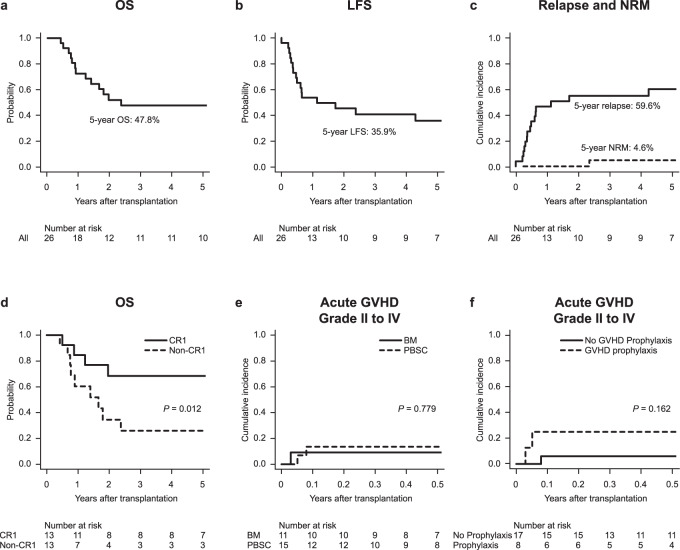
Transplant outcomes in the syngeneic group. **a** Overall survival (OS) rate, **b** leukemia-free survival (LFS) rate, and **c** cumulative incidence of relapse and non-relapse mortality (NRM) in the syngeneic (Syn) group. **d** OS stratified by disease status at hematopoietic stem cell transplantation. **e** Cumulative incidence of grade II–IV acute graft-versus-host disease (GVHD), stratified by graft source. **f** Cumulative incidence of grade II–IV acute GVHD, stratified by GVHD prophylaxis status. *BM* bone marrow, *CR1* first complete remission, *PBSC* peripheral blood stem cell.

The 5-year OS and LFS rates and cumulative incidence rates of relapse and NRM were 47.8% (95% confidence interval [CI], 27.5–65.7%; Fig. 2a), 35.9% (95% CI, 17.6–54.6%; Fig. 2b), 59.6% (95% CI, 36.7–76.5%; Fig. 2c), and 4.6% (95% CI, 0.3–19.9%; Fig. 2c), respectively. The 5-year OS rate was significantly higher in patients in CR1 (68.4%; 95% CI, 35.9–86.8%) than that in patients in non-CR1 (26.0%; 95% CI, 6.3–51.7%; *P* = 0.012; Fig. 2d). No significant differences in OS rates were observed after stratifying patients according to age (*P* = 0.404), sex (*P* = 0.250), cytogenetic risk (*P* = 0.175), graft source (*P* = 0.489), or the year of HSCT (*P* = 0.404).

The median number of days from HSCT to neutrophil engraftment was 13.5 (95% CI, 9–20). There was no significant difference after stratifying by graft source (BM, 17.5 days [95% CI, 10–20 days] vs. PBSC, 11.0 days [95% CI, 9–14 days]; *P* = 0.062). The median number of days from HSCT to platelet engraftment was 26.0 days (95% CI, 9–284 days). There was no significant difference after stratifying by graft source (BM, 26.0 days [95% CI, 16–37 days] vs. PBSC, 17.0 days [95% CI, 9–284 days]; *P* = 0.055).

The 100-day cumulative incidence rate of grade II acute GVHD was 11.5% (95% CI, 2.8–27.1%). No patient developed grade III–IV acute GVHD. There was no significant difference after stratifying by graft source (BM, 9.1% [95% CI, 0.4–34.7%] vs. PBSC, 13.3% [95% CI, 2.0–35.5%]; *P* = 0.779; Fig. 2e) or GVHD prophylaxis (with prophylaxis, 25.0% [95% CI, 3.0–57.9%] vs. without prophylaxis, 5.9% [95% CI, 0.3–24.3%]; *P* = 0.162; Fig. 2f). Acute GVHD occurred in 1 of 11 (9.1%) patients receiving syngeneic PBSCT without GVHD prophylaxis.

The 1-year cumulative incidence rate of chronic GVHD was 4.3% (95% CI, 0.3–19.0%). There was no significant difference after stratifying by graft source (BM, 0.0% [95% CI, 0.0–0.0%] vs. PBSC, 7.7% [95% CI, 0.3–31.7%]; *P* = 0.921) or GVHD prophylaxis (with prophylaxis, 14.3% [95% CI, 0.3–51.3%] vs. without prophylaxis, 0.0% [95% CI, 0.0–0.0%]; *P* = 0.137). Chronic GVHD was not observed in patients receiving syngeneic PBSCT without GVHD prophylaxis.

In the Syn group, 14 (53.8%) patients died. Non-relapse death occurred in only one (3.8%) patient. This patient died from an infection. Among 13 (50.0%) patients after relapse, the causes of death were disease progression (*n* = 6, 23.1%), infection (*n* = 3, 11.5%), organ failure (*n* = 3, 11.5%), and interstitial pneumonia (*n* = 1, 3.8%). No patient for whom the data were available (*n* = 24, 92.3%) developed secondary cancer.

### Comparison of outcomes among the Syn, Auto, MSD, and MUD groups

Regarding comparison cohorts, 476, 1755, and 1222 patients in CR1 met the inclusion criteria in the Auto, MSD, and MUD groups, respectively. Of these, 39 patients per group and 13 patients in the Syn group were selected after PS matching. The C-statistic of the PS model was 0.713 (95% CI, 0.587–0.846), 0.775 (95% CI, 0.668–0.882), and 0.798 (95% CI, 0.697–0.899) between the Syn and Auto, MSD, MUD groups, respectively. These values indicated acceptable discrimination. PS matching created comparable cohorts balanced in terms of age at HSCT, sex, cytogenetic risk, and the year of HSCT (Table 2).

**Table 2:** Patient characteristics after propensity score matching.

	Syn		Auto		MSD		MUD		Syn vs. Auto		Syn vs. MSD		Syn vs. MUD	
Total number	13		39		39		39		*P*	SMD	*P*	SMD	*P*	SMD
Age at HSCT									1.000	<0.01	1.000	<0.01	1.000	<0.01
≥40 years	7	(53.8%)	21	(53.8%)	21	(53.8%)	21	(53.8%)						
Sex									1.000	<0.01	1.000	<0.01	1.000	<0.01
Male	9	(69.2%)	27	(69.2%)	27	(69.2%)	27	(69.2%)						
Cytogenetic risk									1.000	<0.01	1.000	<0.01	1.000	<0.01
Favorable	2	(15.4%)	6	(15.4%)	6	(15.4%)	6	(15.4%)						
Intermediate	10	(76.9%)	30	(76.9%)	30	(76.9%)	30	(76.9%)						
Poor	0	(0.0%)	0	(0.0%)	0	(0.0%)	0	(0.0%)						
Unevaluable	1	(7.7%)	3	(7.7%)	3	(7.7%)	3	(7.7%)						
Disease status									1.000	<0.01	1.000	<0.01	1.000	<0.01
CR1	13	(100.0%)	39	(100.0%)	39	(100.0%)	39	(100.0%)						
Graft source									<0.01	1.118	1.000	<0.01	<0.01	1.789
BM	5	(38.5%)	0	(0.0%)	15	(38.5%)	39	(100.0%)						
PBSC	8	(61.5%)	39	(100.0%)	24	(61.5%)	0	(0.0%)						
Year of HSCT									1.000	<0.01	1.000	<0.01	1.000	<0.01
2004–2017	7	(53.8%)	21	(53.8%)	21	(53.8%)	21	(53.8%)						

*Auto* autologous, *BM* bone marrow, *CR1* first complete remission, *HSCT* hematopoietic stem cell transplantation, *MSD* matched sibling donor, *MUD* matched unrelated donor, *PBSC* peripheral blood stem cell, *SMD* standardized mean difference, *Syn* syngeneic.

**Fig. 3:**
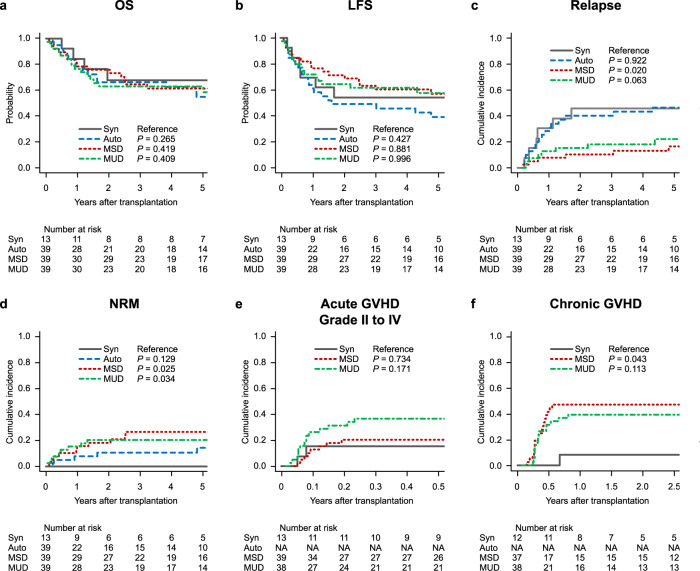
Transplant outcomes according to the donor group. **a** Overall survival (OS) rate. **b** Leukemia-free survival (LFS) rate. **c** Cumulative incidence of relapse, **d** non-relapse mortality (NRM), **e** grade II–IV acute graft-versus-host disease (GVHD), and **f** chronic GVHD. *Auto* autologous, *MSD* matched sibling donor, *MUD* matched unrelated donor, *Syn* syngeneic.

Transplant outcomes per donor group are shown in Fig. 3. The 5-year OS rates after HSCT were 68.4% (95% CI, 35.9–86.8%; reference) in the Syn group, 55.9% (95% CI, 37.2–71.0%; *P* = 0.265) in the Auto group, 62.4% (95% CI, 44.8–75.8%; *P* = 0.419) in the MSD group, and 63.7% (95% CI, 46.5–76.7%; *P* = 0.409) in the MUD group (Fig. 3a). The 5-year LFS rates after HSCT were 53.8% (95% CI, 24.8–76.0%; reference) in the Syn group, 38.6% (95% CI, 22.6–54.5%; *P* = 0.427) in the Auto group, 56.6% (95% CI, 39.0–70.9%; *P* = 0.881) in the MSD group, and 57.1% (95% CI, 39.4–71.5%; *P* = 0.996) in the MUD group (Fig. 3b).

The 5-year cumulative incidence rate of relapse in the Syn group (46.2%; 95% CI, 17.8–70.7%; reference) was significantly higher than that in the MSD group (16.7%; 95% CI, 6.6–30.9%; *P* = 0.020), was higher than that in the MUD group (22.2%; 95% CI, 10.0–37.5%; *P* = 0.063) groups but not statistically significant, and was comparable to that in the Auto (46.9%; 95% CI, 29.5–62.5%; *P* = 0.922) group (Fig. 3c). The 5-year cumulative incidence rate of NRM in the Syn group (0.0%; 95% CI, 0.0–0.0%; reference) was significantly lower than those in the MSD (26.7%; 95% CI, 13.7–41.6%; *P* = 0.025) and MUD (20.6%; 95% CI, 9.5–34.6%; *P* = 0.034) groups but not significantly different from that in the Auto group (14.4%; 95% CI, 5.0–28.6%; *P* = 0.129) (Fig. 3d).

The 100-day cumulative incidence rates of grade II–IV acute GVHD were 15.4% (95% CI, 2.2–39.8%; reference) in the Syn group, 20.6% (95% CI, 9.5–34.6%; *P* = 0.734) in the MSD group, and 36.8% (95% CI, 21.7–52.0%; *P* = 0.171) in the MUD group (Fig. 3e). The 1-year cumulative incidence rate of chronic GVHD in the Syn group (8.3%; 95% CI, 0.4–32.8%; reference) was significantly lower than that in the MSD group (47.2%; 95% CI, 30.1–62.6%; *P* = 0.043) but not significantly different from that in the MUD group (39.5%; 95% CI, 23.9–54.7%; *P* = 0.113) (Fig. 3f).

## Discussion

The present study examined factors affecting outcomes after syngeneic HSCT for patients with AML. Although previous studies on syngeneic HSCT mainly included patients receiving syngeneic BMT [17–20], more than half of patients received syngeneic PBSCT in the present study. Our results of survival, acute and chronic GVHD, and engraftment rates were not significantly different between syngeneic BMT and PBSCT. In allogeneic HSCT, PBSCT is associated with rapid hematopoietic recovery, but controversies remain regarding survival, relapse, NRM, and GVHD risks [31–35]. A nationwide study on patients with AML after allogeneic HSCT by Yanada et al. [35] showed that related PBSCT was associated with the risks of grade III–IV acute GVHD, chronic GVHD, and NRM higher than those associated with BMT. Both RFS and OS rates were poorer for related PBSCT than for related BMT. In the present study, the number of cases was too small to draw a conclusion regarding syngeneic HSCT. The choice of graft source should be determined carefully, considering both recipient- and donor-related factors.

Fouillard et al. [20] reported that the diagnosis of acute GVHD was made for 8 of 22 (36.4%) patients with GVHD prophylaxis and 11 of 140 (7.9%) patients without GVHD prophylaxis, although statistical significance was not assessed. In the present study, the rates of acute and chronic GVHD were not significantly different between patients with and without GVHD prophylaxis. Although the previous report mainly included patients receiving BMT [20], in the present study, more than half of patients received syngeneic PBSCT. Only one (9.1%) and no patient receiving PBSCT without GVHD prophylaxis developed grade II acute GVHD and chronic GVHD, respectively. Our findings raise the question of whether GVHD prophylaxis is necessary for syngeneic HSCT even if PBSC is used as a stem cell source.

Although Gale et al. [17] compared the outcomes of syngeneic BMT with those of allogeneic BMT from MSD in patients with AML, acute lymphoblastic leukemia (ALL), and chronic myeloid leukemia, the present study focused exclusively on patients with AML in CR1 to compare the outcomes of syngeneic HSCT with those of allogeneic HSCT from MSD or MUD. The OS and LFS rates in the Syn group were similar to those in the MSD and MUD groups. The relapse rate in the Syn group was higher than that in the MSD and MUD groups and was offset by a lower NRM. Given the present findings, syngeneic HSCT might be feasible as an alternative post-remission treatment for patients with AML in CR1.

According to the recent survey of the European Society of Blood and Marrow Transplantation, the use of autologous HSCT for AML has decreased over several years [36]. On the other hand, nationwide studies on patients with AML in CR1 by Mizutani et al. [11, 12] reported autologous PBSCT remains a viable alternative as post-remission therapy in patients with AML in CR1. They showed that autologous PBSCT was associated with lower NRM and higher relapse rates than those associated with allogeneic PBSCT or BMT from MSD and allogeneic BMT from MUD, resulting in comparable OS and LFS rates. The present study is the first to compare the outcomes of syngeneic HSCT with those of autologous PBSCT. The OS, LFS, relapse, and NRM rates in the Syn group were not significantly different from those in the Auto group. The priority of the procedures remains unclear from our study. The advantage of syngeneic over autologous HSCT is that the infused hematopoietic stem cells are free from damage from cytotoxic chemotherapy and the risk of leukemic contamination [18]. Nevertheless, clinicians must carefully consider donor-associated risk, unlike in autologous HSCT [37–39]. Further studies are needed to distinguish the roles of syngeneic and autologous HSCT procedures in patients with AML.

There are several limitations to the present study owing to its registry-based retrospective nature. First, the sample size was small because of the rarity of syngeneic HSCT. We were unable to identify significant differences in the outcomes of syngeneic HSCT based on factors other than disease status owing to the small sample size. Second, little information was available on the number of chemotherapy cycles and the dose of nucleated cells infused. Barrett et al. [18] reported that the dose of nucleated cells infused affected survival in patients with AML, ALL, and chronic myeloid leukemia after syngeneic BMT. Fouillard et al. [20] reported that the number of induction courses to reach CR1 affected outcomes after syngeneic BMT or PBSCT for patients with AML and ALL. Third, we could not obtain data on minimal residual disease (MRD) or mutational profiles. Previous studies demonstrated that MRD at the time of HSCT predicted relapse and survival in patients with acute leukemia [40–43]. The presence of high-risk molecular markers, such as *FLT3-ITD* or *TP53* mutations, has been associated with poor prognosis in allogeneic HSCT [44, 45]. These factors may have differed among our cohorts, potentially affecting outcomes. Fourth, no data on zygosity diagnosis could be obtained using DNA analyses [46]. It is possible that some patients who received HSCT from a dizygotic twin were included in the Syn group, although we confirmed monozygosity based on the compatibility of sex, HLA, and ABO blood type. Fifth, we could not get the information on other candidate donors of each patient owing to a lack of data. We do not know whether there were patients who had identical twins but chose other donors. Sixth, we could not add the conditioning regimen to the PS factors because the present study had a small number of patients in the Syn group. In addition, in six (23.1%) patients of the Syn group, we could not get the information on the dose of the conditioning drugs owing to old data. There is a possibility that the conditioning regimen might influence our results. Therefore, the present findings should be interpreted with caution. Nevertheless, the present study is worth reporting because of the rarity of patients with AML undergoing syngeneic HSCT.

In conclusion, this is the first study to assess prognostic factors in patients with AML after syngeneic HSCT and compare the outcomes of syngeneic HSCT with those of autologous or allogeneic HSCT for patients in CR1. Our findings suggest that syngeneic HSCT might offer an alternative curative option for AML. Data from a larger number of patients and prospective studies are needed to clarify the role of syngeneic HSCT in the treatment of AML.

## Supplementary information

Reproducibility checklist
